# The effect of health anxiety on attitudes toward disease prevention in individuals with and without a family history of hypertension

**DOI:** 10.1038/s41371-025-01036-2

**Published:** 2025-06-24

**Authors:** Yasemin Karacan, Ayşe Gül Parlak

**Affiliations:** 1 Yalova University, Faculty of Health Sciences, Department of Nursing, Yalova, Türkiye; 2 Kafkas University, Faculty of Health Sciences, Department of Nursing, Kars, Türkiye

**Keywords:** Risk factors, Renovascular hypertension

## Abstract

This study aims to examine how health anxiety and demographic characteristics influence attitudes toward hypertension prevention in individuals with and without a family history of hypertension (family HHT). This cross-sectional study included 1,139 individuals over the age of 18. Data were collected through an online survey and analyzed using the Attitudes Scale Towards Prevention of HT (ASPH) and the Health Anxiety Scale (HAS). In addition to scale scores, demographic characteristics and family history of hypertension were also collected. The survey link was shared via social media, allowing participants to distribute it within their networks. The mean ASPH score was 98.31 ± 21.91, highest in prevention and control (31.55 ± 7.28). Age correlated with mental and physical activity (p = 0.026) and nutritional behavior (p = 0.041), while BMI was linked to hypertension attitudes (p = 0.006). Regression analysis showed that gender (B = −6.609, p < 0.001) and a family HHT (B = −0.574, p = 0.013) significantly influenced attitudes toward hypertension prevention, with men and those with a family HHT scoring lower than their counterparts. Scatter plots revealed higher health anxiety and stronger hypertension attitudes in those with hypertensive parents, while attitudes were more varied among individuals with hypertensive siblings or grandparents. These findings highlight the impact of family hypertension history and demographics on hypertension attitudes and health anxiety, emphasizing early awareness, targeted health education for gender differences, and preventive strategies for high-risk groups.

## Introduction

Hypertension is defined as a sustained systolic blood pressure (SBP) >140 mmHg and/or diastolic blood pressure (DBP) >90 mmHg in the International Society of Hypertension (ISH) guidelines. Hypertension is associated with an increased risk of mortality and cardiovascular events such as coronary heart disease, heart failure, and stroke [1]. An estimated 1.5 billion people worldwide have high blood pressure, but only 14% of them are under control [2]. Hypertension, which often progresses without obvious symptoms and causes serious health complications, is known as the “silent killer” worldwide [3, 4]. It is estimated that globally around 46% of adults with hypertension are unaware of their disease [5]. Particularly in low- and middle-income countries, the hypertension awareness rate is one-third, and low awareness can lead to untreated progression of the disease. Systolic blood pressure >115 mmHg accounted for an estimated 8.5 million deaths in 2015, 88% of which occurred in low- and middle-income countries [6]. Hypertension is one of the leading risk factors for early death worldwide, causing approximately 1.8 million preventable deaths and 235 million years of life lost or years resulting in disability each year (Disability Adj. Life Years, DALYs) [7].

The prevalence of hypertension, a common health condition in Turkey, varies between 30.3% and 36.5% in the adult age group, increasing with age and reaching 70-80% in the elderly population [8]. Despite implementing serious health policies and practices for the prevention of hypertension in cooperation with the WHO [9], hypertension awareness in Turkey is still insufficient [8, 10–13].

Family history of hypertension (family HHT) indicates that genetic factors have a major role in the development of the disease and that these individuals have a higher risk of hypertension given their genetic predisposition. Previous studies in the literature [14–16] highlighted that individuals with a family HHT have a higher awareness of the disease. In a study conducted by Mirzaei et al. [4] in Iran, it was determined that 80.2% of individuals with a family HHT had an awareness of the disease and that these individuals were more willing to manage the disease and practice preventive health behaviors [4]. Research on the effect of individuals with a family HHT on their attitudes toward the prevention of the disease In Turkey is limited [17].

Nurses play a critical role in identifying the impact of demographic factors and family history associated with hypertension on individuals’ attitudes, enhancing awareness, and developing new strategies aimed at more informed efforts in disease prevention [18]. Chronic diseases negatively impact the psychological well-being of not only the patient but also their family members. The presence of a chronic disease in the family may increase the risk of developing health anxiety in family members, which in turn may affect their attitudes and behaviors toward hypertension [19].

Health anxiety is essential for building individual awareness and healthy attitudes in the fight against hypertension, as it encourages more vigilance against HAS-2, notably hypertension [20]. In this regard, nurses assume a critical role in raising awareness in individuals with hypertension, providing the necessary information for disease management, and promoting healthy living behaviors. In individuals diagnosed with hypertension, nursing care provides regular blood pressure monitoring, access to suitable treatment, and encouragement of lifestyle changes to ensure blood pressure control, increase compliance with treatment, and contribute to maintaining quality of life [18, 21, 22].

Health anxiety may influence attitudes toward hypertension prevention in individuals with a family history of the condition, making it an important factor to consider when developing effective prevention strategies [23]. This study may contribute to healthcare delivery and public health policies by analysing the role of health anxiety and attitudes on hypertension awareness of individuals. Therefore, this study aims to examine the effect of health anxiety and demographic characteristics on hypertension prevention attitudes in individuals with and without a family HHT.

## Methods

### Study design

This descriptive and cross-sectional study was conducted between October 3, 2024, and January 30, 2025. The study population consisted of adults over 18 years old living in Turkey who had the ability to complete surveys electronically. Convenience sampling was employed to reach a broad audience quickly, easily, and economically [24]. A total of 1307 individuals initially participated in the survey. After excluding responses with missing data, outliers, logical inconsistencies, invalid answers to scale items, and unrealistically short completion times, 1139 participants were included in the final analysis. The survey was designed so that submission was only possible after completing all items, minimizing missing data. The online survey was created using Google Forms and distributed through the researchers’ personal social media accounts (e.g., WhatsApp, Instagram, Facebook). Participants were encouraged to further share the link within their own social networks, including family members, friends, and close contacts. Because of the open and self-directed nature of recruitment, it was not possible to determine the total number of individuals who viewed the survey.

Ethical approval was obtained from the Non-Interventional Clinical Research Ethics Committee of the Yalova Univertsity where the study was conducted, with the approval dated Oct. 2, 2024, and numbered 219. The study was conducted in accordance with the Declaration of Helsinki. In the first section of the online data collection form, participants were informed about the study, its purpose was explained, and a consent button was included.

### Measurements

The online data collection form consisted of three sections. The first section included informed consent. The second section featured a “Personal Information Form,” while the third section employed the “Attitudes Scale Towards Prevention of HT” and the “Health Anxiety Scale.”

### Personal information form

The form, developed by the researcher based on the literature, contained 13 questions covering demographic information (age, gender, marital status, place of residence, income, employment status, smoking and alcohol consumption, height, weight, etc.) and questions about family HHT and blood pressure monitoring.

### Attitudes scale towards prevention of HT (ASPH)

Developed by Albayrak and Şengezer (2022) for the Turkish population, the ASPH is a 26-item scale validated for reliability. The scale consists of five subdimensions: prevention and control, habits and lifestyle, nutritional attitude, mental state and physical activity, and disease and risk awareness. Each item is rated on a 5-point Likert scale from “Strongly Disagree” to “Strongly Agree,” with scores ranging from 26 to 130. There is a positive correlation between attitudes towards hypertension prevention and scale scores. The ASPH addresses risk factors for hypertension that individuals can mitigate and allows them to self-assess their risk of illness. The overall reliability (Cronbach’s alpha) of the scale is greater than 0.9, [17].

### Health anxiety scale (HAS)

The Health Anxiety Scale (HAS) was developed by Salkovskis et al. [25] to assess health-related anxiety, and its validity and reliability for the Turkish population were established by Aydemir et al. [26]. The HAS is an 18-item self-report scale, where 14 items use a four-point ordinal response format to evaluate participants' mental state, while the remaining 4 items assess their emotional response to the belief that they have a serious illness. The scale includes two sections: (1) the core section (HAS total), which measures overall health anxiety, and (2) the supplementary section, which assesses the perceived negative consequences of illness (HAS-2) (4 items). Each item is scored from 0 to 3, with higher scores indicating greater health anxiety. The reliability analysis of the scale demonstrated a high internal consistency, with a Cronbach’s alpha coefficient of 0.918 [25].

### Data analysis

This study was analysed using the SPSS 20.0 software (IBM SPSS Statistics for Windows). Descriptive statistics (frequency, percentage, etc.) and frequency distributions were obtained for the study data. For continuous variables, the mean, standard deviation, median, and interquartile range (IQR) were calculated. The normality of continuous variables was tested using the Kolmogorov-Smirnov test. Where data were not normally distributed, distributions were reported using the median and IQR. The direction and strength of the effects of independent variables on hypertension attitude and health anxiety were determined by Spearman Correlation Analysis. Multiple linear regression analysis was conducted to examine the effects of demographic variables and HAS on ASPH and its subdimensions. In this analysis, the dependent variables included the total ASPH score and its subdimensions (prevention and control, nutritional attitude, habits, and lifestyle), while the independent variables included age, gender, marital status, educational level, income, family HHT and scores from the HAS total and HAS-2 subdimension. Additionally, scatter plots with grouping were used to visualize the distribution of HAS total, HAS-2, and ASPH scores based on the presence or absence of a family HHT.

## Results

### Participants characteristics and their impact on hypertension attitudes

An analysis of participants' demographic characteristics showed a median age of 29 years, with 51.0% of participants between 18-29 years old. Of the participants, 67.5% were female (p = 0.002), 6.8% were single (p = 0.032), 62.7% were university graduates (p = 0.059), and 65.8% lived in province (p = 0.523). In terms of income, 38.8% reported sufficient income, 3.7% had income equivalent to expenses, and 3.5% stated their income was insufficient (p = 0.971). Employment-wise, 45.4% were unemployed, with no significant association observed (p = 0.268). Among lifestyle factors, 26.8% reported smoking (p = 0.555) and 9.3% reported alcohol consumption (p = 0.215). Regarding family HHT, 45.2% had a family history, with 16.5% having a history in their mother and 11.7% in their grandparents (p = 0.001), indicating a statistically significant association. Additionally, 53.5% monitored their blood pressure, while 46.5% did not (p = 0.924), showing no significant difference (Table 1).

**Table 1:** Descriptive characteristics of participants and their impact on hypertension attitudes.

Characteristics (n = 1139)		n	%	Statistics	p
Age	18-29 30-41 42-53 54-65	581 271 200 87	51.0 23.8 17.6 7.6	H (χ²) = 2.825	0.419
Place of residence	Province District Village/town	749 311 79	65.8 27.3 6.9	H (χ²) = 1.297	0.523
Gender	Male Female	370 769	32.5 67.5	Z = −3.090 U = 126209.0	0.002**
Marital status	Married Single Single (Spouse death/devorced)	400 692 47	35.1 60.8 4.1	H (χ²) = 6.865	0.032*
Education status	Primary School Secondary Education High School University	34 129 262 714	3.0 11.3 23.0 62.7	H (χ²) = 7.444	0.059
Income status	Covering expenses Does not cover expenses Equivalent to expenses	442 347 350	38.8 30.5 30.7	H (χ²) = 0.058	0.971
Employment status	Not working Pensioner Public Private sector Student	517 49 166 218 189	45.4 4.3 14.6 19.1 16.6	H (χ²) = 5.190	0.268
Smoking	Yes No	305 834	26.8 73.2	Z = −0.591	0.555
Alcohol consumption	Yes No	106 1033	9.3 90.7	Z = −1.239 U = 50756.0	0.215
Hypertension in the family	Mother Father Sibling Child Grandparents Aunt-uncle Spouse No	188 108 19 2 133 40 25 624	16.5 9.5 1.7 0.2 11.7 3.5 2.2 54.8	H (χ^2^) = 24.715	0.001**
Blood pressure monitoring	Yes No	609 530	53.5 46.5	Z = −0.096 U = 160856.5	0.924
BMI median: 23.7, IQR: 6.2 Age median:29.0, IQR: 20					

Interquartile Range (IQR). body mass index (BMI).

According to the **IQR = Q3 - Q1** formula, the difference between the third quartile (Q3) and the first quartile (Q1) in the data is 6.2 units; indicating a range spread over 6.2 units. p < 0.05*, p < 0.01**, U = Mann-Whitney U, H (χ^2^) = Kruskal-Wallis H.

Table 2 shows the total and subdimensions of ASPH and total and HAS. The mean total score of ASPH was found to be 98.31 ± 21.91, and the mean scores for prevention and control, disease and risk knowledge, mental state and physical activity, habits and lifestyle, and nutritional attitude were 31.55 ± 7.28, 19.28 ± 4.62, 10.18 ± 2.42, 23.41 ± 5.56, and 13.89 ± 3.37, respectively. The mean scores for the HAS total were found to be 13.75 ± 6.81 in the HAS total and 3.50 ± 2.34 in the HAS-2.

**Table 2:** Distribution of mean total and subdimension scores of participants in ASPH and HAS.

Scales and subdimension		Mean ± SD	min	max
ASPH	Prevention and control	31.55 ± 7.28	8.0	40.0
	Disease and risk information	19.28 ± 4.62	5.0	25.0
	Mental state and physical activity	10.18 ± 2.42	3.0	15.0
	Habits and lifestyle	23.41 ± 5.56	6.0	30.0
	Nutrition behaviour	13.89 ± 3.37	4.0	20.0
	ASPH total	98.31 ± 21.91	26.0	130.0
HAS	HAS total	13.75 ± 6.81	1.0	42.0
	HAS-2	3.50 ± 2.34	0.0	27.0

*ASPH* attitudes scale towards prevention of HT, *HAS* health anxiety scale, *HAS total* hypersensitivity to physical symptoms and anxiety, *HAS-2* perception of negative consequences of disease.

According to the results of the Spearman correlation analysis, a significant positive correlation was found between age and BMI (r = 0.261, p = 0.000), mental state and physical activity (r = 0.066, p = 0.026), and nutritional behavior (r = 0.061, p = 0.041). BMI showed a positive correlation with disease and risk information (r = 0.065, p = 0.039) and HAS-2 (r = 0.086, p = 0.006). Regarding ASPH subdimensions, disease and risk information had strong positive correlations with mental and physical activity (r = 0.590, p = 0.000), prevention and control (r = 0.831, p = 0.000), habits and lifestyle (r = 0.800, p = 0.000), nutritional behavior (r = 0.644, p = 0.000), and total attitude (r = 0.911, p = 0.000). Similarly, mental and physical activity was positively correlated with prevention and control (r = 0.518, p = 0.000), habits and lifestyle (r = 0.527, p = 0.000), nutritional behavior (r = 0.619, p = 0.000), and total attitude (r = 0.681, p = 0.000). Prevention and control showed strong positive correlations with habits and lifestyle (r = 0.835, p = 0.000), nutritional behavior (r = 0.632, p = 0.000), and total attitude (r = 0.913, p = 0.000). Additionally, habits and lifestyle had positive correlations with nutritional behavior (r = 0.624, p = 0.000) and total attitude (r = 0.896, p = 0.000), while nutritional behavior was positively correlated with total attitude (r = 0.785, p = 0.000). For health anxiety, a moderate positive correlation was found between HAS total and HAS-2 (r = 0.453, p = 0.000) (Table 3).

**Table 3:** Correlation analysis of the relationship between hypertension attitudes, demographic characteristics, and health anxiety (1,2).

Variables		Age	BKI	Disease and risk information	Mental state and physical activity	Prevention and control	Habits and lifestyle	Nutrition behaviour	Attitude total	HAS total	HAS-2
Age	r	1.000	0.261**	0.056	0.066^*^	0.012	0.018	0.061^*^	0.043	0.013	0.009
	p		**0.000**	0.060	**0.026**	0.685	0.549	**0.041**	0.145	0.659	0.749
BMI	r	0.261**	1000	0.065*	0.043	0.094**	0.051	0.046	0.086**	0.020	0.009
	p	0.000		**0.039**	0.173	**0.003**	0.105	0.140	**0.006**	0.521	0.780
Disease and risk information	r	0.056	0.065*	1.000	0.590^**^	0.831^**^	0.800^**^	0.644^**^	0.911^**^	0.046	0.053
	p	0.060	**0.039**		**0.000**	**0.000**	**0.000**	**0.000**	**0.000**	0.123	0.076
Mental state and physical activity	r	0.066^*^	0.043	0.590^**^	1.000	0.518^**^	0.527^**^	0.619^**^	0.681^**^	0.048	0.032
	p	**0.026**	0.173	**0.000**		**0.000**	**0.000**	**0.000**	**0.000**	0.105	0.278
Prevention and control	r	0.012	0.094**	0.831^**^	0.518^**^	1.000	0.835^**^	0.632^**^	0.913^**^	0.034	0.040
	p	0.685	**0.003**	**0.000**	**0.000**		**0.000**	**0.000**	**0.000**	0.260	0.182
Habits and lifestyle	r	0 .018	0.051	0.800^**^	0.527^**^	0.835^**^	1.000	0.624^**^	0.896^**^	0.036	0.035
	p	0.549	0.105	**0.000**	**0.000**	**0.000**		**0.000**	**0.000**	0.221	0.242
Nutrition behaviour	r	0.061^*^	0.046	0.644^**^	0.619^**^	0.632^**^	0.624^**^	1.000	0.785^**^	0.022	00.001
	p	**0.041**	0.140	**0.000**	**0.000**	**0.000**	**0.000**		**0.000**	0.469	0.966
Attitude total	r	0.043	0.086**	0.911^**^	0.681^**^	0.913^**^	0.896^**^	0.785^**^	1.000	0.052	0.043
	p	0.145	**0.006**	**0.000**	**0.000**	**0.000**	**0.000**	**0.000**		0.078	0.150
HAS total	r	0.013	0.020	0.046	0.048	0.034	0.036	0.022	0.052	1.000	0.453^**^
	p	0.659	0.521	0.123	0.105	0.260	0.221	0.469	0.078		**0.000**
HAS-2	r	0.009	0.009	0.053	0.032	0.040	0.035	0.001	0.043	0.453^**^	1.000
	p	0.749	0.780	0.076	0.278	0.182	0.242	0.966	0.150	**0.000**	

*BMI* body mass ındex.

p < 0.05*, p < 0.01**.

According to the multiple linear regression analysis, a model was constructed to examine the effects of age, gender, marital status, education, employment, blood pressure measurement frequency, family HHT, HAS (total,2) and disease perception on ASPH and its subdimensions. The ASPH total and its subdimensions were the dependent variables, and only statistically significant variables are presented in the model. Gender and family HHT together explained 21% of the variance in the ASPH Total (Adj. R² = 0.21). Gender had a significant negative effect (B = −6.609, p ≤ 0.001), indicating that men had lower hypertension attitude scores compared to women. Family HHT also contributed significantly (B = −0.574, p = 0.013). In the habits and lifestyle subdimension, gender and family HHT together explained 23% of the variance (Adj. R² = 0.23). Gender had a strong negative effect (B = −1.822, p ≤ 0.001), while family HHT had a weaker but still significant impact (B = −0.132, p = 0.024). For the nutrition behaviour subdimension, gender alone explained 12% of the variance (Adj. R² = 0.12). Gender had a significant effect (B = −0.685, p = 0.003). In the mental state and physical activity subdimension, gender alone explained 17% of the variance (Adj. R² = 0.17). Gender had a significant effect (B = −0.656, p ≤ 0.001). For the disease and risk information subdimension, gender and family HHT together explained 22% of the variance (Adj. R² = 0.22). Gender had a significant effect (B = −1.205, p ≤ 0.001), while family HHT also contributed meaningfully (B = −0.146, p = 0.003). In the prevention and control subdimension, gender and family HHT together explained 22% of the variance (Adj. R² = 0.22). Gender had a strong effect (B = −2.241, p ≤ 0.001), and family HHT also played a significant role (B = −0.203, p = 0.008).

**Fig. 1:**
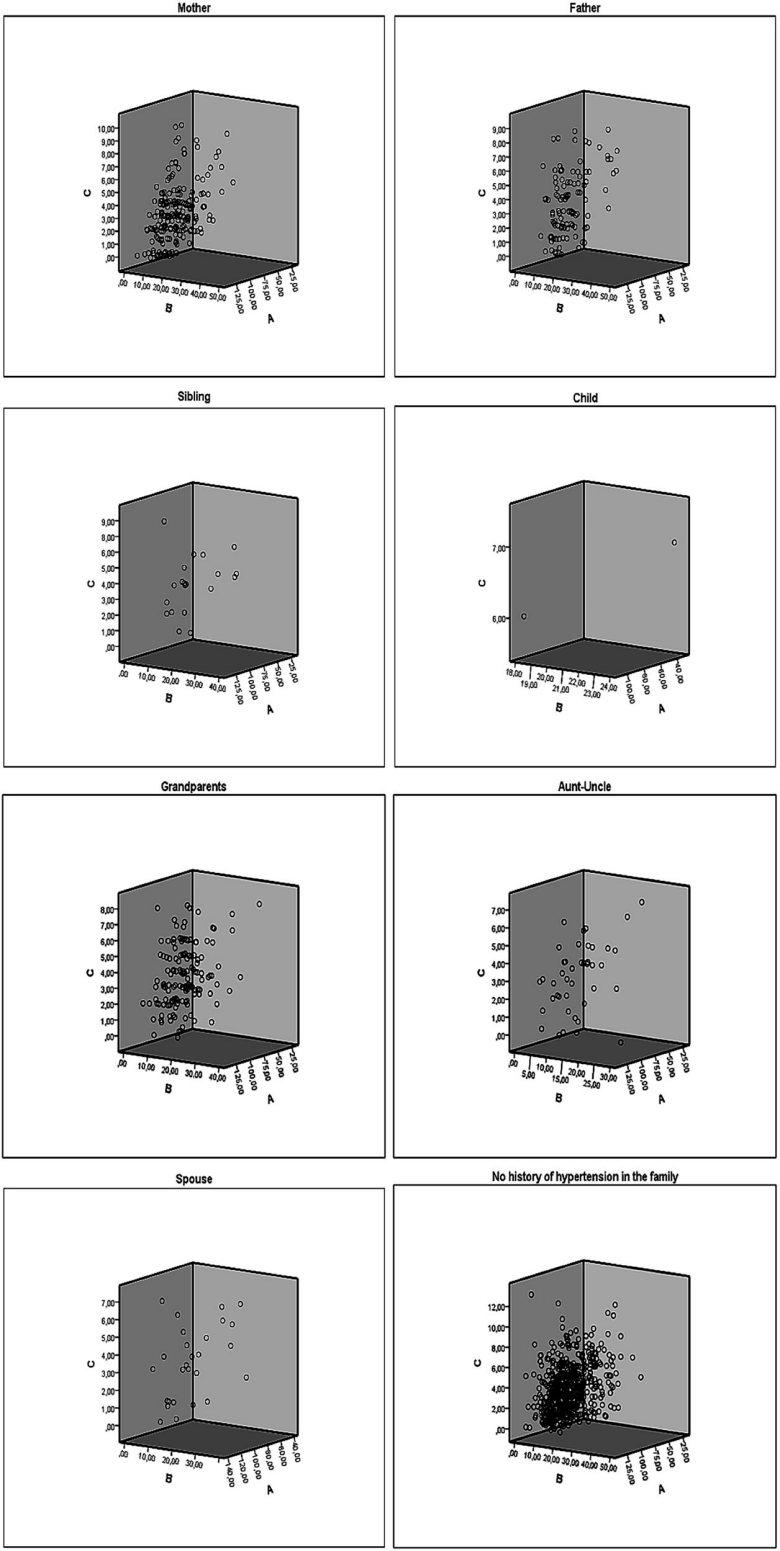
Relationship between health anxiety and attitudes toward hypertension according to family history of hypertension. This figure illustrates the differences in health anxiety dimensions and attitudes toward hypertension prevention based on the presence of a family HHT. **A** Attitudes toward hypertension prevention (ASPH). **B** Hypersensitivity to physical symptoms and anxiety (HAS Total). **C** Perception of negative consequences of illness (HAS-2).

In this study, health anxiety and hypertension attitudes total were examined across eight categories based on the presence of hypertension in family members (mother, father, siblings, children, grandparents, aunts, uncles, and spouse) and visualized using scatter plots with grouping (Fig. 1). Hypertension attitude total (Axis A), Hypersensitivity to physical symptoms and anxiety (HAS total) (Axis B), and HAS-2 (Axis C) were shown. Among individuals with a history of hypertension in their mother and father, heightened levels of HAS total were observed to significantly increase hypertension attitudes, with intensified attitudes among these individuals. For individuals with a history of hypertension in siblings, mid-level distribution of attitudes and anxiety levels was observed, with variance concentrated around the median rather than extremes. While these individuals showed heightened awareness of HAS-2, they did not exhibit excessive sensitivity towards physical symptoms. Due to limited data, it was challenging to identify a general trend among individuals with a child having hypertension. However, based on available data, no statistically significant clustering or concentration between hypertension attitudes and anxiety levels was observed. Although attitudes were more widely distributed among individuals with a history of hypertension in their grandparents, a clear trend was still observed. The attitudes of those with aunts or uncles with hypertension showed a more scattered distribution without a prominent trend. Those with spouses with a hypertension history demonstrated notably high attitudes, appearing more sensitive to the potential negative consequences of the disease. Among individuals without a family HHT, some concentration was observed along certain axes in the figure. On Axis A, representing attitudes towards hypertension, and Axis B, representing HAS total, data points clustered at low to moderate levels, indicating a certain intensity in attitudes and anxiety levels. However, on Axis C, concerning perceptions of negative consequences, data points were more broadly distributed without a clear clustering pattern.

**Fig. 2:**
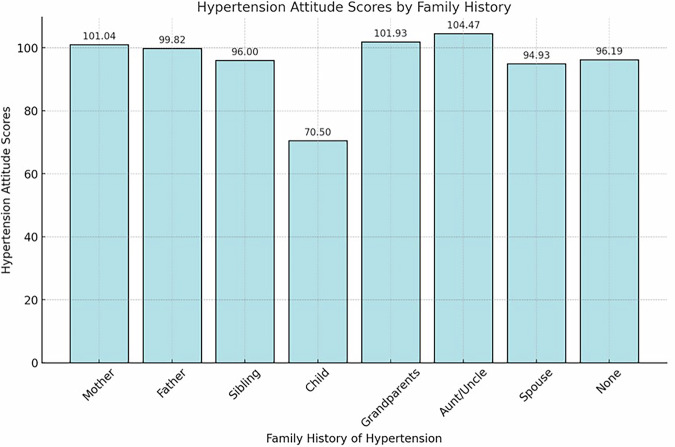
Mean hypertension attitude scores across groups defined by family history of hypertension.

In Fig. 2, the Kruskal-Wallis test and Dunn’s post-hoc analyses revealed statistically significant differences in hypertension attitude scores among individuals with a family history of hypertension (H (χ²) = 24.715, p = 0.001). Specifically, individuals with a maternal (101.04 ± 20.04), paternal (99.82 ± 19.74), sibling (96.00 ± 29.68), grandparent (101.93 ± 20.30), and aunt/uncle (104.47 ± 17.22) history of hypertension exhibited higher hypertension attitude scores, while those with a history of hypertension in children (70.5 ± 47.38) and spouses (94.93 ± 21.90) displayed lower scores. Individuals without a family history of hypertension (96.19 ± 22.88) showed a more varied distribution of scores, indicating a more heterogeneous pattern in attitudes.

## Discussion

This study contributes to the literature by being among the first to examine in detail the impact of a family HHT on health anxiety and related attitudes. By investigating the attitudes toward hypertension prevention in individuals with and without a familial risk of hypertension, this study primarily aimed to examine the impact of health anxiety on these attitudes, while also seeking to identify the effect of demographic characteristics and family history as a secondary objective.

The educational background of participants suggests a generally high level of health literacy, which is known to positively affect disease perception and health attitudes [18, 26]. Furthermore, the presence of financial strain among a portion of participants highlights the potential influence of socioeconomic status on individuals’ ability to engage in preventive health behaviours. This result aligns with the data reported by Schutte et al. [6] that the highest mortality rates standardized by income level and age in 2019 were in low-middle income and low-income countries [6]. Particularly in Turkey, a low-income country in the Middle East, these risks are also relevant to us, and this is believed to increase the pressure on our healthcare system. Uncontrolled hypertension and other health problems thus pose a major threat to public health and are considered a source of understandable concern.

45.2% of the participants reported a family HHT. This rate is higher than the 31.2% family history reported by Arslantaş et al. [13]. Similarly, a study by Kanuda et al. (2018) on hypertension awareness reported a family history rate of 48.2% [14]. Shah et al. [27], in a study investigating a family HHT in Abu Dhabi, found that 2.9% of children had a family HHT [28]. These results suggest that genetic factors are more prevalent in certain communities. Additionally, differences in our study's results may be attributed to the participants' demographic characteristics, socioeconomic conditions, and regional factors.

In Table 2, the prevention and control scores (31.55 ± 7.28) in the subdimensions of the family ASPH indicate high awareness among individuals regarding hypertension prevention. A study by Paulose et al. [29] in southern Ethiopia found that individuals exhibited moderate attitudes toward controlling and preventing hypertension. However, disease and risk knowledge remained lower at 62.7% [27], as in this study, indicating gaps in understanding hypertension risk factors and effects within our country. Thus, it is evident that more informative content on hypertension risks is needed in public health programs.

In controlling hypertension, lifestyle modifications such as dietary changes, reducing alcohol consumption, quitting smoking, and aerobic exercise have now broadened to include less obvious strategies like stress reduction, isometric exercise, and reducing exposure to air pollution [29]. In this study, it is notable that individuals scored average on mental state, physical activity, and nutritional attitude scales. Ma et al. reported that only 32.3% of hypertension patients engaged in physical activity, with emotional stability maintained in only 3.1% and a sad mood in 10.9% [30]. In a 2017 study by Son et al., 15.8% of hypertensive patients who did not engage in regular physical activity experienced depressive symptoms. Zhu and Wang (2024) demonstrated that individuals who eat healthily and stay physically active have a 25% lower likelihood of developing hypertension compared to those with unhealthy diets and inactive lifestyles [31]. Additionally, in this study, high scores in lifestyle and habit factors were observed, with a healthy weight and diet respectively reducing the risk of hypertension by 54% and 28% [32], making these results encouraging. Although the ASPH total score (98.31 ± 21.91) was high, physical activity and mental state are often overlooked in hypertension management, indicating a need for increased awareness and educational efforts in these areas [23].

Health anxiety concerning HAS total (13.75 ± 6.81) was low, suggesting that participants generally showed low sensitivity to physical symptoms and did not experience excessive anxiety. Compared to Bulut and Bozo's (2022) study, health anxiety and greater sensitivity to physical symptoms were reported in individuals with chronic diseases [33]. However, the low anxiety level of individuals in their study may be attributed to factors such as the variability of health awareness and risk perception of different sample groups and the fact that the patient group was not diagnosed with hypertension. Furthermore, very low scores for HAS-2 (3.50 ± 2.34) suggest that individuals are less concerned about the long-term effects of hypertension. This contrasts with the results of Havill et al. [21] and suggests that there is a need for awareness-raising by nurses regarding long-term outcomes.

The overall hypertension control rate is lower among young adults compared to middle-aged or elderly adults [34]. In the correlation analysis conducted, age was significantly associated with some subdimensions of hypertension attitude. A positive correlation was found between age and mental state and physical activity (p = 0.026) and age and nutritional behavior (p = 0.041), indicating that older individuals tend to adopt more active and health-conscious behaviors. Additionally, BMI showed a positive correlation with total attitude (p = 0.006), indicating that individuals with higher BMI scores may exhibit stronger hypertension-related attitudes. This suggests that individuals with a higher BMI might be more conscious of hypertension risks and adopt more proactive attitudes toward disease prevention and management. However, in the regression analysis, men were shown to have lower hypertension attitude scores than women, indicating that women demonstrate higher awareness and more cautious attitudes toward hypertension. The higher awareness of hypertension in women and their more cautious attitudes may be due to their greater sensitivity to health risks, as frequently mentioned in the literature. Similarly, Zhang and Moran (2017) found that individuals aged 18-39 exhibited lower attitudes toward hypertension compared to those aged 40 and above, with young women displaying higher hypertension awareness than men in that age range [34]. Conversely, in a study by Gong et al. [35], age was identified as an important factor in attitudes toward hypertension, showing that younger individuals exhibited more positive attitudes [36]. However, no significant difference was found between men and women regarding hypertension attitude. These differences in results may be attributed to cultural, socioeconomic, and geographic variations. Particularly, societal health perceptions and access to healthcare services can impact individuals’ attitudes toward chronic conditions like hypertension. This may explain why men are less likely to seek healthcare services and pay less attention to health-related matters [37].

It is often noted that women are more conscious than men in matters related to diet and health behaviors, which plays a significant role in managing chronic diseases such as hypertension [38, 39]. The regression analysis showed that gender had a significant effect on nutritional attitudes (B = −0.685, p ≤ 0.001), consistent with the literature[34, 40, 41]. The lower nutritional attitudes among men may stem from a lower level of motivation or awareness in adopting healthy eating habits [42]. Furthermore, demographic factors such as marital status, educational level, and income level did not significantly impact hypertension attitudes, suggesting that these variables do not strongly influence hypertension attitudes. Effective hypertension management generally involves individuals consciously taking preventive and control measures [43]. The literature also supports that prevention and control strategies are critical in managing hypertension [14, 35, 43]. It is also believed that the prevention and control of hypertension are associated with lifestyle factors [29]. In our model, the high constant coefficient for habits and lifestyle (Table 4) reflects changes in hypertension attitude. Similarly, Perdita et al. [44] identified a link between lifestyle habits and hypertension awareness among the adult population [45]. The high constant coefficient for habits and lifestyle in our model suggests that lifestyle factors significantly impact hypertension attitudes. Nursing care plays a crucial role in assisting individuals to adopt these lifestyle changes, encouraging healthy habits, and providing effective strategies for hypertension management. This result is supported in the literature by studies emphasizing the interconnectedness of lifestyle and hypertension prevention strategies [43, 45], indicating that lifestyle changes and preventive health behaviors are essential in managing hypertension.

**Table 4:** The impact of independent variables on ASPH total: regression analysis results.

Dependent variable/ Model	Independent variable	Un Std. Coeff		Std. Coeff	t	p	95% CI
		B	Std. Error	Beta			Lower/upper
ASPH total R² = 0.29 Adj. R² = 0.21	Constant	111.846	8.349		13.397	0.000	95.465/128.226
	Gender	−6.609	1.472	−0.141	−4.488	**0.000**	−9.498/−3.720
	Family HHT	−0.574	0.231	−0.075	−2.487	**0.013**	−1.027/−0.121
Habits and lifestyle R² = 0.32 Adj.R² = 0.23	Constant	26.080	2.116		12.323	0.000	21.927/30.233
	Gender	−1.822	0.373	−0.154	−4.881	**0.000**	−2.554/−1.089
	Family HHT	−0.132	0.058	−0.068	−2.264	**0.024**	−0.247/−0.018
Nutrition behaviour R² = 0.20 Adj.R² = 0.12	Constant	17.323	1.291		13.422	0.000	14.791/19.856
	Gender	−0.685	0.228	−0.095	−3.007	**0.003**	−1.131/−0.238
Mental state and physical activity R² = 0.25 Adj. R² = 0.17	Constant	11.676	0.923		12.644	0.000	9.865/13.488
	Gender	−0.656	0.163	−0.127	−4.029	**0.000**	−0.976/−0.332
Disease and risk information R² = 0.30 Adj. R² = 0.22	Constant	21.142	1.758		12.026	0.000	17.693/24.591
	Gender	−1.205	0.310	−0.122	−3.887	**0.000**	−1.813/−0.597
	Family HHT	−0.146	0.049	−0.090	−3.002	**0.003**	−0.241/−0.051
Prevention and control R² = 0.31 Adj.R² = 0.22	Constant	35.624	2.772		12.852	0.000	30.186/41.063
	Gender	−2.241	0.489	−0.144	−4.584	**0.000**	−3.200/−1.282
	Family HHT	−0.203	0.077	−0.0080	−2.653	**0.008**	−0.354/−0.053

The multicollinearity analysis revealed that for all models, the Tolerance values for Gender and Family HHT were 0.868 and 0.954, respectively, while the VIF values were 1.152 and 1.048, respectively. These values indicate that there is no multicollinearity issue among the independent variables.

*UnStd. Coeff* unstandardized coefficients *Std. Coeff* standardized coefficients, *95% CI* 95% confidence interval.

Although an overall negative association was observed between family HHT and attitudes toward hypertension prevention, subgroup analyses revealed that individuals with hypertensive parents or siblings exhibited more favourable attitudes, as indicated by their ASPH scores. These findings suggest that close familial ties may enhance individuals’ health awareness and concern regarding hypertension. Furthermore, regression analyses identified both family HHT and gender as significant predictors of hypertension-related attitudes. Notably, men demonstrated lower levels of awareness and less engagement in preventive behaviors, which is consistent with previous research [28, 46, 47]. However, a study conducted in Sudan reported low disease awareness among those with a family HHT, indicating the potential moderating role of sociocultural and demographic factors [48]. Moreover, individuals with hypertensive spouses or children reported higher anxiety and stronger disease-related attitudes (see Fig. 1), possibly due to emotional closeness and perceived vulnerability. In support of this, Kubb and Foran [49] found that health anxiety is often shared within families, with a significant correlation between anxiety levels for spouses and children. Expanding on subgroup-level findings, the regression analysis supported a similar pattern in the habits and lifestyle subdimension of hypertension attitudes. Gender appeared to have a stronger negative impact on this subdimension compared to family HHT. In this study, individuals with hypertensive spouses demonstrated higher anxiety and stronger disease-related attitudes, consistent with Hu’s (2022) hypothesis that social and emotional bonds amplify disease perception [50]. This finding may be explained by the perception of illness as a personal threat due to the closeness and interdependence of the spousal relationship. For individuals with hypertensive children, attitudes and anxiety levels varied more widely. This heterogeneity may reflect how parental concerns differ individually, leading to varied emotional responses and perceptions. Similarly, participants with hypertensive grandparents showed more variable attitudes. This is consistent with Priboi et al. (2016), who noted that grandparents often view themselves as emotional anchors in the family, prioritizing the well-being of others over their own [51].

In a study by Wakefield et al. (2014) with the grandparents of children with cancer, it was similarly shown that these individuals minimized their emotional and physical needs in order to stay strong for their families [22]. These results may be explained by elderly individuals’ insufficient sharing of health information within the family or a decline in health awareness over time, which can prevent the transmission of this knowledge to their grandchildren. In the study by Havill et al. [21], it was shown that living with a sibling who has a chronic illness can cause health anxiety in healthy siblings, leading them to worry about their own health. Compared to that study, individuals with a family HHT in their siblings were observed to have moderate levels of anxiety and attitudes toward their own health in our study. According to Havill’s results, individuals might be expected to feel more anxiety regarding their health, while the present study indicates that their attitudes toward hypertension are more balanced and less extreme [21]. This suggests that, unlike other chronic illnesses, hypertension may result in a more balanced level of concern regarding one’s health, or that individuals, despite being aware of the adverse effects of the disease, may not exhibit HAS total. In Herrera et al.’s [42] study, it was shown that pushing individuals with chronic conditions such as diabetes and hypertension to comply with treatment, through pressure and comparison (using an aunt as an example), had adverse effects [52].

In a quantitative study by Bulut and Bozo [34] on individuals with health anxiety, the loss of a cousin due to cancer and the recurrence of an aunt’s illness were identified as significant predictors of elevated health anxiety levels. Unresolved loss issues, particularly, were central to participants' health anxiety and showed indications of hypochondriasis [33]. In this study, however, individuals with an aunt, uncle, or other extended family members with a history of illness showed a more scattered pattern in their health attitudes compared to those with immediate family members. This suggests that the health history of more distant relatives may have a more indirect and ambiguous effect on the individual. While the health history of close relatives, such as a parent or sibling, tends to result in more pronounced changes in health attitudes, a history of hypertension in more distant relatives, such as an aunt or uncle, did not lead to clear attitudes in the individual. This result indicates that the influence of family members may vary depending on their closeness and that a history of illness in distant relatives may lead to more complex attitudes. Examining Fig. 1, it is suggested that a family HHT intensifies individuals’ awareness and attitudes toward hypertension, while those without such a history may hold a broader and more varied perception. Additionally, although our regression analysis did not reveal a direct effect of HAS total and HAS-2 on ASPH, Fig. 1 suggests a relationship between family HHT and both HAS total and HAS-2 scores. This suggests that, indirectly, increased health anxiety in individuals with a family HHT may influence their hypertension attitudes. The finding that a family history of close relatives with hypertension leads to significant changes in anxiety and attitudes, whereas more distant relatives’ histories have a more ambiguous effect, offers a new perspective in the fields of internal medicine, public health, and nursing care.

## Limitations

This study has several limitations. Since it was conducted via an online survey, only individuals with internet access could participate, which limits the generalizability of the results. Due to the cross-sectional design, causality between variables could not be assessed. Since the data were self-reported, there are limitations regarding the accuracy of the responses. The majority of participants were university graduates, which may make it difficult to generalize the results to individuals with lower educational levels.

## Conclusions

Our analysis demonstrated that gender and family HHT significantly influence hypertension attitudes, with men showing lower awareness and weaker preventive behaviors. The findings emphasize the need for gender- and knowledge-based educational programs led by nurses to enhance hypertension awareness and promote healthier lifestyle choices. Targeted interventions, particularly for individuals with a family HHT, can play a crucial role in fostering preventive health behaviors and reducing associated risks, highlighting the essential contribution of nursing care in hypertension management.

## Summary

### What is known about this topic

- Hypertension is a major public health concern globally, with familial history being a significant risk factor.
- Health anxiety and demographic characteristics influence individuals' attitudes and behaviors toward hypertension prevention and management.
- Gender differences in hypertension awareness and attitudes are well-documented, with women typically demonstrating higher awareness than men.

### What this study adds

- In Turkey, hypertension is a prevalent health issue; however, awareness, treatment, and blood pressure control remain inadequate.
- Men have lower hypertension awareness and participate less in preventive behaviors compared to women.
- A family history of hypertension increases disease awareness, with the highest awareness observed in individuals whose mother has a history of hypertension.

## Data Availability

The datasets generated and/or analysed during the current study are available from the corresponding author, Dr. Yasemin Karacan, upon reasonable request.
